# Prevalence of obesity among adults in Otjomuise Community in Namibia

**DOI:** 10.4102/hsag.v30i0.3074

**Published:** 2025-08-05

**Authors:** Simone Ferreira, Craig Vincent-Lambert

**Affiliations:** 1 Department of Sport and Movement Studies, Faculty of Health Sciences, University of Johannesburg, Johannesburg, South Africa; 2 Department of Emergency Medical Care, Faculty of Health Sciences, University of Johannesburg, Johannesburg, South Africa

**Keywords:** BMI, global epidemic, noncommunicable diseases, community-based interventions, community members

## Abstract

**Background:**

Obesity is a major contributor to non-communicable diseases (NCDs) and is rising in socioeconomically disadvantaged communities with limited health literacy and access to nutritious food. Namibia, like many African countries, faces increasing obesity rates because of dietary and lifestyle transitions. Community-based screening is essential for early identification and prevention.

**Aim:**

This study aimed to determine the prevalence of obesity among community members in Otjomuise, Namibia.

**Setting:**

The study was conducted at a community health care clinic in Otjomuise, Namibia.

**Methods:**

A cross-sectional, descriptive study was conducted with 335 adults. Body mass index (BMI) was calculated and classified according to Centers for Disease Control and Prevention (CDC) categories: underweight, healthy weight, overweight, or obese.

**Results:**

Of the 335 participants, 67.2% (*n* = 225) were female and 32.8% (*n* = 110) male. Overall, 9.3% (*n* = 31) were underweight, 48.7% (*n* = 163) had a healthy weight, 26.6% (*n* = 89) were overweight, and 16.7% (*n* = 56) were obese. Mean BMI was 22.92 ± 4.64 kg/m^2^ for males and 26.76 ± 6.28 kg/m^2^ for females.

**Conclusion:**

Less than half of the participants had a healthy BMI, with notable overweight and obesity prevalence, particularly among women. These findings align with regional and global trends and underscore the need for targeted public health strategies.

**Contribution:**

This study provides updated obesity data for Namibia and supports community-based screening as a tool for surveillance and intervention. It highlights the importance of multisectoral approaches promoting diet, physical activity, and health education to reduce NCD risk.

## Introduction

Obesity remains a significant public health concern worldwide, with rising prevalence rates particularly notable in low- and low-middle-income countries (World Obesity Atlas 2024). Namibia, like many developing nations, is experiencing an increasing burden of obesity, which contributes to a range of noncommunicable diseases (NCDs) such as diabetes, cardiovascular disease (CVD), hypertension and certain cancers (Kazembe, Nickanor & Crush 2022). The World Health Organization (WHO) reports that globally, obesity has nearly tripled since 1975, with 2.5 billion adults aged 18 years and older classified as obese (WHO 2024a). In sub-Saharan Africa, obesity rates are escalating, primarily driven by rapid urbanisation, lifestyle changes and economic development (Wang et al. 2025). In 2019, higher-than-optimal body mass index (BMI) caused an estimated 5 million deaths from NCDs such as CVD, diabetes, cancers, neurological disorders, chronic respiratory diseases and digestive disorders (Global Burden of Disease Collaborative Network 2020).

The urbanisation process in sub-Saharan Africa, including Namibia, is associated with a shift towards sedentary lifestyles and increased consumption of western diets that are commonly characterised by energy-dense, nutrient-poor foods (Mensah 2021). This epidemiological transition has resulted in a dual burden of malnutrition, where undernutrition coexists with overnutrition (Nel & Steyn 2022). Urban residents are more likely to engage in behaviours that increase obesity risk, such as high-calorie diets, physical inactivity and the consumption of processed foods (Nashandi et al. 2024).

Obesity is influenced by various factors, including genetic predisposition, environmental influences and socio-economic status (Ghosh et al. 2023). In peri-urban settings like Otjomuise, a low-income area in Namibia, limited access to healthy foods, inadequate opportunities for physical activity and socio-economic challenges contribute to higher obesity rates (Crush, Nickanor & Kazembe 2020). Recent surveys indicate that obesity prevalence in Namibia’s urban populations is increasing, with estimates suggesting that over 30% of adults are obese (The Namibia Ministry of Health and Social Services [MoHSS] and ICF International 2014). Despite the growing obesity epidemic, reliable and up-to-date data on obesity prevalence in Namibia remains scarce. The Namibia Demographic and Health Survey (NDHS) reported that urban females (40% prevalence) are more likely to be overweight or obese compared to rural females (22% prevalence), but the last NDHS was published in 2013, and that is the only national data available (Nashandi, Monyeki & Reilly 2024; Ndishishi 2014).

Establishing accurate obesity rates is crucial for understanding its extensive impact on the general population. Obesity significantly increases the risk of developing NCDs leading to higher morbidity and mortality rates (Mohajan & Mohajan 2023). According to the WHO, NCDs are responsible for 74% of deaths globally, with a substantial proportion attributed to conditions associated with obesity (WHO 2024b). The economic burden of obesity is substantial, contributing to rising health care costs, reduced workforce productivity and increased absenteeism (Dall et al. 2024). In the United States, for instance, obesity-related health care costs are estimated at nearly $173bn annually (Nagi et al. 2024).

Beyond physical health, obesity also impacts mental well-being, often being associated with depression, low self-esteem and social stigma. The co-occurrence of health burdens in transitioning populations, particularly in specific socioeconomic and cultural contexts, exacerbates these challenges (Cruz-Ávila et al. 2024). In low-income communities, where health care access is often limited (Abdul et al. 2024), the consequences of obesity can be even more severe, exacerbating health inequalities and straining public health systems. The WHO notes that poverty is closely linked with NCDs, and the rapid rise in NCDs is predicted to impede poverty reduction initiatives in low-income countries, particularly by increasing household costs associated with health care (WHO 2024b).

Understanding obesity prevalence is therefore essential for implementing targeted interventions that improve overall population health and well-being. Early identification and intervention are crucial to mitigate the health impacts of obesity. Effective strategies include community-based health promotion, increased access to affordable healthy foods and the creation of environments that support physical activity. This study investigated the prevalence of obesity among community members in Otjomuise, a low-income, peri-urban area of Namibia.

## Research methods and design

### Study design

A cross-sectional, descriptive, quantitative research design was adopted.

### Setting

Data were gathered from adult community members in Otjomuise, Namibia who presented themselves for free health screening during a community outreach project. The Otjomuise government Primary Health Care (PHC) clinic is located within the community of Otjomuise, with the nearest government hospital, Katatura Hospital in Khomas being 10 km away (Statista n.d.). The data collection took place just outside the Otjomuise PHC clinic, within the clinic grounds. Otjomuise is a peri-urban community on the outskirts of Windhoek, Namibia. According to the most recent census data, the population of Otjomuise is estimated to be approximately 67 211 residents (Statista n.d.). The township is bordered by other residential areas such as Katutura and Hakahana, which together form a large urban cluster within Windhoek. This community is characterised by poverty, substance abuse, high unemployment rates and scarce resources such as access to good food and health care services (Adewunmi et al. 2023).

### Study population and sampling strategy

Participants consisted of community members of Otjomuise, aged 18 years and older, who provided written consent for their demographic and health data to be gathered as part of the health screenings. Individuals who presented themselves at the PHC clinic were invited to participate in the health screenings, as part of the research project. The participants were included if they resided in Otjomuise, were over the age of 18 years and gave written consent. The community was purposively chosen by the Department of Clinical Health Sciences at the Namibia University of Science and Technology (NUST). Because of the closest hospital being 10 km away, the researchers and academics selected this region to provide free health screenings for the community, to allow access to health care services. They were also selected because of low levels of knowledge on healthy lifestyles and physical activity habits (Nashandi et al. 2024).

### Data collection

This research was part of a greater outreach project, arranged by the University of Johannesburg (UJ) and NUST. The outreach project was aimed at assisting the local clinic in screening individuals for NCDs and related comorbidities. Individuals who were identified as overweight or obese received educational advice and guidance by the trained research team after the testing was concluded. Screening stations were erected at the government PHC clinic in Otjomuise, which allowed privacy of each individual participant during the screenings. Community members in the surrounding area and those attending the clinic were invited for health screenings over the course of 2 days. The screenings were conducted by trained students and staff from the two universities, who underwent rigorous training to ensure consistency on the screening protocols. Each participant’s data were recorded on individual data-collection sheets and thereafter, the data were captured on a Microsoft Excel^®^ spreadsheet for analysis. Data were gathered which included gender, age, highest level of education, ethnic group, marital status, employment status, household size and average annual earning per household.

The screening process also included focused history-taking, knowledge on various risk factors for different NCDs and measurement of physical parameters such as height, weight and calculation of BMI. While various additional parameters and tests were conducted, this paper focuses solely on the analysis and findings pertaining to datasets relevant to the prevalence of obesity within the sample.

Participants had their weight measured using a Micro Life^®^ diagnostic scale placed on an even surface, while their height was measured using a standard wall-mount stadiometer. All equipment utilised were calibrated and verified before and during every data-collection day and were kept in one place for the entire data-collection day. Participants were required to remove their shoes and personal items for these measurements. Using the measured heights and weights, the BMI for each participant was calculated. The formula used for the calculation of BMI is weight (kg)/height (m)^2^. With the metric system, the formula for BMI is weight in kilograms divided by height in meters squared. Because height is commonly measured in centimetres, height in centimetres was divided by 100 to obtain height in meters. Calculated BMIs were then categorised according to the Centres for Disease Control and Prevention (CDC) categories as either ‘underweight’, ‘healthy weight’, ‘overweight’ or ‘obese’ (refer to Table 1).

**TABLE 1:** Body mass index classification according to the CDC (2015).

BMI	Weight status
Below 18.5 kg/m^2^	Underweight
18.5 kg/m^2^ – 24.9 kg/m^2^	Healthy weight
25.0 kg/m^2^ – 29.9 kg/m^2^	Overweight
30.0 kg/m^2^ and above	Obesity

BMI, body mass index.

In instances where participants could not communicate in English, translators from Namibia, who were also students and staff from NUST, were able to communicate in their native language, relay information and ensure effective communication with these participants. This ensured that none of the participants were excluded from the study based on their language proficiency.

### Data analysis

The data collected were entered into a Microsoft Excel^®^ spreadsheet. Socio-economic data were initially recorded in separate columns, followed by columns for weight and height, enabling analysis of BMI.

Statistical analysis was conducted using IBM SPSS version 26.0 software. Descriptive statistics including frequencies and percentage were used to summarise the distribution of the data. A Chi-square test of independence was conducted to examine the relationship between gender (male, female) and BMI category (Underweight, Healthy weight, Overweight, Obesity Class 1, Obesity Class 2, Obesity Class 3). For further analysis of gender distribution across BMI categories, post-hoc z-tests for proportions were conducted, with Bonferroni corrections applied to adjust for multiple comparisons. Additionally, mean and standard deviation of the male and female participants were conducted separately to summarise the central tendency and dispersion of BMI values. To assess the distributional characteristics of BMI by gender, skewness statistics were computed to quantify the degree of asymmetry in each group’s BMI distribution. A positive skewness value indicates a distribution with a longer right tail, reflecting a tendency toward higher BMI values. The significance level was set at *p* < 0.05.

The classification of obesity is arranged according to the BMI categories, set out by the CDC (2015), namely *underweight, healthy weight, overweight* and *obesity* (Class 1, Class 2 and Class 3). The resulting BMI value allows for a standardised comparison across different populations and is utilised extensively in both clinical and research settings to evaluate the risk of developing weight-related health conditions.

Referring to the categories of obesity as indicated in Table 1, in this study, participants were considered to have ‘abnormal’ weight status when their BMI was greater than 25 kg/m^2^, indicating that they are overweight. Obesity Class 1 was noted as a BMI of 30 kg/m^2^ – 34.9 kg/m^2^, Obesity Class 2 as a BMI of 35 kg/m^2^ – 39.9 kg/m^2^ and Obesity Class 3 as a BMI of ≥ 40 kg/m^2^.

### Ethical considerations

The study received approval from the UJ’s Research Ethics Committee (Ethical Clearance Number REC-1985–2023). Permission was also obtained from the Namibian MoHSS and the Faculty of Health, Natural Resources and Applied Sciences of NUST to conduct the study. Additionally, permission was sought from the head of the local health clinic to engage with community members. Prior to conducting the screening, written consent was obtained from each participant, and a unique identifier was assigned to maintain participant anonymity and ensure privacy protection.

## Results

The sample consisted of 335 participants, where 67.2% (*n* = 225) were female and 32.8% (*n* = 110) were males. As indicated in Table 2, the 18–29 age group was the most represented by the male participants consisting of 25.5% (*n* = 28) and the 40–49 age group by the female participants comprising 26.7% (*n* = 62). Only 1.8% of the participants (*n* = 6) were over the age of 70. Only 19.4% of the participants (*n* = 65) reported to have completed high school and less than half of the participants reported to be employed or self-employed (*n* = 145), while 43.6% (*n* = 146) reported to be unemployed. Most of the participants, 65.7% (*n* = 220), were never married.

**TABLE 2:** Summary of participants’ demographic and socioeconomic data.

Variable	Frequencies (*n*)		Percentage	
	Males (*n* = 110)	Females (*n* = 225)	Males	Females
**Age (years)**				
18–29	28	53	25.50	23.6
30–39	19	49	17.37	21.8
40–49	23	62	20.90	27.6
50–59	22	45	20.00	20.0
60–69	16	12	14.50	5.3
70–79	2	1	1.80	0.4
80–89	0	3	0.00	1.3
**Level of education**				
No formal schooling	11	15	9.70	6.2
Less than primary school	5	60	4.40	24.7
Primary school	36	13	31.60	5.4
Secondary school	32	70	39.10	31.1
High school	18	47	15.80	19.3
Tertiary education	6	17	5.30	7.0
Postgraduate degree	0	2	0.00	0.8
Refused to answer	2	1	1.80	0.4
Total	110	225	-	-
**Occupation**				
Government employee	1	20	0.90	9.8
Non-government employee	20	42	18.50	20.6
Retired	7	1	6.50	0.5
Student	11	25	10.20	12.3
Self-employed	24	38	22.20	18.6
Unemployed (unable to work)	13	13	12.00	6.4
Unemployed (able to work)	34	86	30.90	38.2
Total	110	225	-	-
**Marital status**				
Cohabitating	2	4	1.80	1.8
Currently married	29	49	26.40	21.8
Divorced	5	2	4.60	0.9
Never married	69	151	62.70	67.1
Separated	4	8	3.60	3.6
Widowed	1	11	0.90	4.9
Total	110	225	-	-

### Prevalence of obesity

In Table 3, underweight (BMI below 18.5 kg/m^2^): 11.8% of the male participants (*n* = 13) and 8.0% of the female participants (*n* = 18) were defined as underweight. Healthy weight (BMI between 18.5 kg/m^2^ and 24.9 kg/m^2^): 63.6% of the males (*n* = 70) and 41.3% of the females (*n* = 93) were categorised as having a normal BMI. Overweight (BMI between 25 kg/m^2^ and 29.9 kg/m^2^): 20.9% of the male participants (*n* = 23) and 29.3% of the female participants (*n* = 66) were classified as overweight. Obesity Class 1 (BMI between 30 kg/m^2^ and 34.9 kg/m^2^): 5.5% of the males (*n* = 6) and 9.3% of the females (*n* = 21) were identified in the Obesity Class 1 category. Obesity Class 2 (BMI between 35 kg/m^2^ and 39.9 kg/m^2^): none of the male participants were categorised in this category and 8.4% of the female participants (*n* = 19) were classified as Obesity Class 2. Obesity Class 3 (BMI ≥ 40 kg/m^2^): Only 0.9% of the males (*n* = 1) and 2.2% of the females (*n* = 5) were categorised as Obesity Class 3. These results conclude that 48.7% of the population has a healthy weight, whereas 42.1% are overweight or obese.

**TABLE 3:** Body mass index classification of 335 participants according to CDC (2015) categories.

Obesity category	BMI	Frequencies (*n*)			Percentage		
		Males	Females	Total	Males	Females	Total
Underweight	Below 18.5 kg/m^2^	13	18	31	11.8	8.0	9.3
Healthy weight	18.5 kg/m^2^ – 24.9 kg/m^2^	70	93	163	63.6	41.3	48.7
Overweight	25.0 kg/m^2^ – 29.9 kg/m^2^	23	66	89	20.9	29.3	26.6
Obesity Class 1	30 kg/m^2^ – 34.9 kg/m^2^	6	21	27	5.5	9.3	8.1
Obesity Class 2	35 kg/m^2^ – 39.9 kg/m^2^	0	19	19	0.0	8.4	5.7
Obesity Class 3	≥ 40 kg/m^2^	1	5	6	0.9	2.2	1.8

BMI, body mass index.

A Chi-square test of independence was performed based on the BMI distribution according to gender (Table 3), to determine whether there was a significant association between gender (male, female) and BMI category (Underweight, Healthy weight, Overweight, Obesity Class 1, Obesity Class 2, Obesity Class 3). The results revealed a statistically significant association between gender and BMI category, χ^2^(5, *N* = 335) = 21.65, *p* = 0.0006.

To explore these differences further, post-hoc *z*-tests for proportions were conducted, with Bonferroni corrections applied to adjust for multiple comparisons (Table 4). The results indicated that a significantly higher proportion of males fell within the healthy weight category compared to females (*z* = 3.47, adjusted *p* = 0.0031). Conversely, a significantly higher proportion of females were classified in Obesity Class 2 than males (*z* = –3.20, adjusted *p* = 0.0082). No statistically significant gender differences were observed in the underweight, overweight, Obesity Class 1 or Obesity Class 3 categories after adjusting for multiple comparisons (all adjusted *p* > 0.05). These findings suggest notable gender-specific trends in body weight distribution, particularly in the healthy and Class 2 obesity ranges.

**TABLE 4:** Distribution of body mass index categories by gender and post-hoc comparisons.

BMI category	BMI	*Z*-Statistic	*p*	Adjusted *p*
Underweight	Below 18.5 kg/m^2^	1.0142	0.3105	1.0000
Healthy weight	18.5 kg/m^2^ – 24.9 kg/m^2^	3.4722	0.0005	0.0031
Overweight	25.0 kg/m^2^ – 29.9 kg/m^2^	−1.8369	0.0662	0.3974
Obesity Class 1	30 kg/m^2^ – 34.9 kg/m^2^	−1.3192	0.1871	1.0000
Obesity Class 2	35 kg/m^2^ – 39.9 kg/m^2^	−3.2020	0.0014	0.0082
Obesity Class 3	≥ 40 kg/m^2^	−0.8921	0.3723	1.0000

BMI, body mass index.

### Body mass index distribution by gender

The mean BMI for male participants was 22.92 ± 4.64 kg/m^2^, whereas females exhibited a higher mean BMI of 26.76 ± 6.28 kg/m^2^.

**FIGURE 1:**
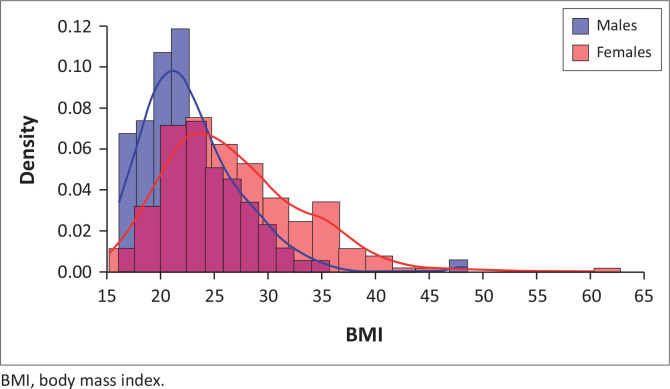
Histogram with Kernel Density Estimate showing the distribution of body mass index among male and female participants (*N* = 335).

Examination of the BMI distributions in Figure 1 revealed a slight positive skewness in both genders, indicating that many individuals fall below the mean with a longer tail extending towards higher BMI values. Notably, the distribution for females was shifted to the right compared to that of males, reflecting a higher central tendency and suggesting that female participants tended to have greater BMI values on average.

Furthermore, the female BMI distribution demonstrated a broader range and greater variability, as evidenced by the larger standard deviation and the presence of more extreme values at the upper end of the spectrum. This increased spread suggests a wider diversity in body composition among females within the sample population. The right-skewness was more pronounced in females, with a longer and heavier tail extending towards higher BMI levels, indicating a subset of females with substantially elevated BMI measurements relative to the male cohort.

Examination of observed and expected frequencies indicated that males were more likely to fall into the healthy weight category, while females were more frequently classified in the overweight and obesity categories than would be expected if the variables were independent.

## Discussion

As indicated in previous literature, obesity and NCDs are on the rise globally and more specifically in communities and contexts characterised by socio economic challenges (Bhuiyan et al. 2024). Africa is a continent with many regions and countries where such challenges prevail (Agyemang et al. 2023), Namibia being no exception.

The findings of this study indicate that many of the participants were either overweight or obese, highlighting a significant public health concern. Obesity rates are generally higher in rural areas compared to urban areas. In the United States, the prevalence of obesity among rural adults was 39.6%, significantly higher than the 33.4% observed in urban adults (Befort, Nazir & Perri 2012). Similarly, in Canada, rural residents had a higher obesity prevalence (30.3%) compared to urban residents (25.2%) (Quan et al. 2022). However, a study conducted from 2014 to 2017 in the Ivory Coast, reported that 83.3% of the participants were obese, with central abdominal obesity affecting 87.2% (Sable et al. 2020). This prevalence aligns with global trends reported by the WHO, which noted that NCDs accounted for 73.9% of all deaths in 2019, with obesity being a major risk factor for NCDs such as CVDs, diabetes and certain cancers (WHO 2024c). This said, differences were noted between the levels of obesity based on gender with more female participants reported in the overweight and/or obese categories that their male counterparts.

Although the findings indicate a high prevalence of overweight and obesity among community members, it is noteworthy that several individuals classified within the normal weight category were found to be on the upper threshold of the normal BMI range, bordering on overweight. This aligns with a study conducted in Nowon Health Care Centre which found that 12% of normal weight individuals were metabolically unhealthy, indicating they might be on the upper threshold of the normal BMI range (Shin et al. 2017). A study conducted in the Peking University community found that individuals with an overweight BMI were significantly more likely to progress to obesity over a 10-year period, suggesting that those at the upper end of the normal weight range are at risk of transitioning to overweight (Guo et al. 2024). While none of the studies explicitly mention participants within the normal weight category being on the upper threshold of the normal BMI range, bordering on overweight, the above studies suggest that individuals on the higher end of the normal BMI range are at risk of transitioning to overweight if preventive lifestyle changes are not adopted. This aligns with the observation that a portion of the normal weight population might be bordering on overweight. Without timely intervention, such as improvements in diet, increases in physical activity, and enhanced health literacy, these individuals may contribute to the growing burden of obesity-related health issues in the community.

### Obesity in females

According to most recent data from a Namibian Demographic and Health Survey published in 2013, the mean BMI of female residents, aged 15–64 was reported to be 23.7 kg/m^2^. At the time of the survey (at a national level) in Namibia, 55.0% of women in this age category were reported to have a healthy weight in relation to their height. In the current study, this percentage was lower, with fewer female participants found to have healthy weights. Reasons for this reduction are unclear but may be related to the specific context of the community the authors engaged with and regardless of the reasons for women of reproductive age, being overweight or obese poses a serious health risk and public health concern (Emmerich et al. 2024). In similar studies conducted in Nigeria and South Africa, the prevalence of overweight and obesity among women was 20.2% and 11.4% in Nigeria, and 28.2% and 44.9% in South Africa, respectively (Akokuwebe & Idemudia 2021). Between 1993 and 2014, the mean BMI among Ghanaian women increased significantly, with obesity projected to affect nearly 23% of women by 2030. It is projected that overweight or obesity would increase to over 35% by 2040 in young women aged 15–24 years in Ghana (Tuoyire 2021).

### Obesity in males

Regarding male participants, the Namibian Demographic and Health Survey found that 65.0% of men, aged 15–64, had a healthy BMI, 23.0% were found to be underweight and 12.0% were classified as overweight or obese. A study showing similar findings was conducted in Ghana, involving 565 Ghanaian adults. They found that the prevalence of overweight and obesity was 29.9% and 22.9%, respectively, with males having higher odds of being of normal weight compared to females (Tsekpetse et al. 2025). The current study found that a greater proportion of male participants had a healthy body weight, which aligns more closely with the findings of the Namibian survey compared to the results observed for women. This is much closer to the findings of the Namibian survey than the current study found in the case of women. The findings from both studies showed that more men tend to have a healthy weight compared to women.

These differences observed between genders can be attributed to various physiological components. At any given BMI, women generally have higher levels of total body fat and lower levels of fat-free mass compared to men. This results in women typically weighing less and being shorter than men (Jayawardena et al. 2020). Additionally, women have higher essential body fat percentages (8% – 12%) compared to men (4% – 6%) (Gao et al. 2025). Women tend to have more gluteo-femoral fat, which is cardioprotective, and less abdominal fat, leading to lower waist–hip ratios compared to men (Olds & Maher 2019). This distribution pattern contributes to different health risks associated with BMI in men and women. These findings highlight gender-specific differences not only in mean BMI but also in the distributional characteristics, suggesting potential implications for targeted health interventions and further investigation into the underlying factors contributing to BMI variability between males and females.

### Impact of obesity

The public health implications of these findings are substantial. Obesity is a known driver of chronic diseases, which accounted for approximately 75.0% of non-pandemic-related deaths globally in 2021 (Giannichi et al. 2024). The increasing prevalence of obesity places a considerable strain on health care systems, leading to higher treatment costs, increased hospitalisations and greater economic burden. For instance, a study estimated that around 1.8bn international dollars would be spent on the direct health care cost of NCDs between 2021 and 2030, primarily because of the continued increase in overweight prevalence. Beyond physical health, obesity is also associated with mental health challenges, including depression, low self-esteem and social stigma, which further diminish the quality of life and well-being (Steptoe & Frank 2023).

These findings further highlight the socioeconomic and environmental factors contributing to obesity. The high prevalence of overweight and obesity observed in this study may be linked to economic disparities, food insecurity and reduced access to safe spaces for physical activity (WHO 2024b). Evidence suggests that low-income communities face a dual burden of malnutrition, where processed, energy-dense foods are more accessible than healthier alternatives. This, combined with sedentary lifestyles, urbanisation and limited health education, creates an environment that promotes obesity (Wells et al. 2020). The WHO emphasises that without targeted interventions, the rising burden of obesity will continue to widen the gap in health inequalities, particularly in vulnerable populations (WHO 2024b).

To address the rising burden of obesity and NCDs, both policy-level and community-based interventions are essential. At the national level, the findings underscore the urgent need for MoHSS to integrate obesity surveillance and prevention into PHC strategies, particularly in urban and peri-urban communities. This aligns with Namibia’s obligations under the WHO Global Action Plan for the Prevention and Control of NCDs (2013–2030), which advocates for multisectoral responses involving nutrition policy, health education and urban design to promote physical activity (Banatvala et al. 2023). Public health strategies should prioritise access to affordable, nutritious food, implement urban planning that encourages physical activity and support educational campaigns focused on healthy lifestyles.

Regionally, the study’s implications extend to policy frameworks such as the African Union’s Africa Regional Nutrition Strategy and the South African Development Community (SADC) Protocol on Health, both of which call for integrated, sustainable approaches to managing nutrition-related health risks (Lokosang, Osei & Covic 2016; Meyer, Wright & Rother 2024). The demonstrated feasibility of community-based screening in Otjomuise can serve as a model for similar socioeconomically challenged urban settings across Southern Africa that are undergoing rapid urbanisation and facing a dual burden of malnutrition. Policymakers across the region could adapt this model to inform context-sensitive prevention programmes, regulate the marketing of unhealthy foods and strengthen local health systems. Ultimately, coordinated national and regional actions are required to reverse current trends and promote healthier populations across sub-Saharan Africa (Meyer et al. 2024).

### International comparisons and broader public health implications

The findings of this study are not unique to Namibia but reflect a wider international pattern, particularly across low- and middle-income countries. Similar trends have been observed in South Africa, Kenya, Brazil and India, where urbanisation, economic inequality and food system transitions have contributed to rising rates of overweight and obesity (Afoakwah, Mahunu & Osei-Kwarteng 2024). In South Africa, the national prevalence of overweight and obesity among adults has surpassed 60%, with women disproportionately affected, paralleling the gender disparity noted in the present study (Boachie et al. 2022; Nglazi & Ataguba 2022). In Kenya, studies report increasing rates of overweight and obesity, particularly in urban populations, with prevalence among women rising to over 30% (Mkuu et al. 2021). In Brazil, nearly 60% of adults are classified as overweight, with rapid increases in obesity linked to dietary shifts and socioeconomic inequality (Motta & Ribeiro 2023). India has experienced a similar dual burden, with urban obesity rates increasing sharply, especially among women, while undernutrition persists in rural areas (Nguyen et al. 2021). These international comparisons underscore the fact that the obesity epidemic is a global issue driven by structural and systemic factors, necessitating cross-border learning and coordinated responses (Popkin, Adair & Ng 2012).

Globally, the public health implications are considerable. The WHO has warned that, if left unchecked, obesity-related conditions will negatively affect health systems, reduce economic productivity and widen health inequalities across nations (WHO 2023). The current findings contribute to this body of evidence, highlighting how urban communities in Namibia mirror global risk trajectories. Multisectoral strategies implemented in other countries, such as taxation on sugary beverages in Mexico (Basto-Abreu et al. 2019), urban planning for active living in the Netherlands (Fishman, Böcker & Helbich 2015) or food labelling reforms in Chile (Pfister & Pozas 2023), offer valuable insights that can be adapted to the Namibian context. Strengthening regional collaborations and participating in global public health initiatives will be key in reversing the rise of NCDs and improving health outcomes across the Global South (WHO 2013).

### Limitations of body mass index in assessing adiposity

While BMI is a practical and widely used tool for estimating obesity prevalence, it has limitations. It does not distinguish between fat and lean mass or indicate fat distribution, which may reduce accuracy in assessing true adiposity, particularly in African populations where body composition patterns vary (Solorzano, Stevens & Doak 2022). Studies have shown regional differences, such as higher obesity rates among South African women compared to West African women and increased central adiposity at lower BMI levels in some African subgroups (Nonterah et al. 2021; Ramsay et al. 2018). These findings highlight the need for context-specific interpretations of BMI. To address this, the current study acknowledges these limitations and recommends future inclusion of complementary measures such as waist circumference or waist-to-height ratio to enhance the assessment of obesity-related risk.

## Conclusion

Obesity has been linked to the development of several NCDs and is on the rise globally, particularly in communities facing socio-economic challenges. Africa, including Namibia, is home to many such contexts. Consistent with findings from other studies, this research identified a high number of overweight and obese individuals within the community, with notable gender differences in obesity levels. Overall, less than half of the participants screened had BMIs within the normal healthy range, with many classified as overweight or obese. This is concerning given the well-established association between obesity and chronic diseases, economic burden and diminished quality of life.

In conclusion, the study confirms the high prevalence of overweight and obesity, affecting a significant portion of the population. If not addressed, this trend could lead to escalating health care costs linked to the management of obesity-related NCDs. Promoting improved health literacy and encouraging healthy lifestyle and dietary habits are key to reversing this pattern. Multi-sectoral strategies that address physical activity, nutrition, and the broader social determinants of health are urgently needed. The implications of this study extend beyond local relevance, offering Namibia an opportunity to contribute to regional and international dialogue on effective obesity prevention strategies. Continued surveillance and updated national data will be essential for evaluating interventions and shaping future public health policies.

### Limitations

Despite the significance of these findings, certain limitations should be acknowledged. This study was conducted within a specific peri-urban population, characterised by unique socioeconomic and environmental factors, including high unemployment, limited access to health care and low health literacy. These contextual determinants can significantly influence lifestyle behaviours, dietary patterns and physical activity levels. As such, the findings may not be generalisable to rural populations, where subsistence lifestyles and different food access patterns prevail, or to higher-income urban areas with better infrastructure and health services. The results are therefore most applicable to similar low-income urban settings undergoing rapid urbanisation and facing a dual burden of malnutrition. Additionally, self-reported dietary and physical activity behaviours were not assessed, which may provide further insights into the underlying causes of obesity in this population.

An inherent limitation of this study design was that the authors relied on community members who arrived for free screening. This meant that the authors were not able to purposefully regulate the sample size, nor could they guarantee a balanced representation of participants across different genders and age groups.

Finally, while the sample size of 335 participants was sufficient for the purposes of the descriptive analysis undertaken for this study, the results and findings may change based on larger sample sizes that include participants from different regions of the country.
